# *Brachionus paranguensis* sp. nov. (Rotifera, Monogononta), a member of the L group of the *Brachionus plicatilis* complex

**DOI:** 10.3897/zookeys.880.28992

**Published:** 2019-10-14

**Authors:** Gerardo Guerrero-Jiménez, Patrizia Elena Vannucchi, Marcelo Silva-Briano, Araceli Adabache-Ortiz, Roberto Rico-Martínez, David Roberts, Roy Neilson, Manuel Elías-Gutiérrez

**Affiliations:** 1 Instituto del Agua, Universidad de Granada, Ramón y Cajal 4, 18071, Granada, España. Departamento de Ecología, Facultad de ciencias, Universidad de Granada, Fuentenueva s/n, 18071Granada, Spain; 2 Centro de Investigación en Ciencias del Mar y Limnología (CIMAR), Universidad de Costa Rica, San Pedro, San José, 11501, Costa Rica; 3 Universidad Autónoma de Aguascalientes, Centro de Ciencias Básicas, Departamento de Biología. Avenida Universidad 940, C.P. 20131, Aguascalientes, Ags. México; 4 Universidad Autónoma de Aguascalientes, Centro de Ciencias Básicas, Departamento de Química. Avenida Universidad 940, C.P. 20131, Aguascalientes, Ags., México; 5 The James Hutton Institute, Craigiebuckler, Dundee DD2 5DA, Scotland, UK; 6 El Colegio de la Frontera Sur, Unidad Chetumal. Av. Centenario Km 5.5, C.P. 77014, Chetumal, Quintana Roo, México

**Keywords:** COI gene, ecology, ITS1, morphometry, rotifers, taxonomy.

## Abstract

The *Brachionus plicatilis* complex represents the most studied group of rotifers, although the systematics of the species complex has not been completely clarified. Many studies have been conducted trying to explore the diversity within the complex, leading to the recognition of three major morphotypes: large (L), small-medium (SM), and small (SS). Currently six species have been described and classified under these types and another nine taxa have been identified but not formally described. Within the L group, three species have been officially described [*B. plicatilis* s.s. (L1), *B. manjavacas* (L2), and *B. asplanchnoidis* (L3)], while a formal description of L4, unofficially known as *B.* ‘Nevada’, is still lacking. In the present study, a new species, *Brachionus paranguensis***sp. nov.**, is formally described and presented as a representative of the L4 clade. The species has been named after a high altitude saline crater lake from Central Mexico, where the specimens were collected. An integrated approach using DNA taxonomy through COI and ITS1 markers, morphology, and ecology was used to confirm the identity of the new species.

## Introduction

The presence of multiple cryptic species that have been classified as a single species due to their morphological similarity still represents a major challenge for biologists (Bickford et al. 2007; Pfenninger and Schwenk 2007). The first approach used when trying to identify and classify an organism is the detection of morphological characters able to distinguish a species. Nevertheless, the exclusive use of morphology-based identification may be problematic when dealing with taxa that lack clear diagnostic characters. To deal with these difficulties, the use of DNA taxonomy represents a valid tool to help reveal the presence of cryptic diversity within taxa whose systematics is still uncertain (Hebert et al. 2004; Hajibabaei et al. 2007). Additionally, complexes of cryptic species may present differences in their ecology, whose characterization might help unravel the identity of such species (Miller 2007).

In rotifers high levels of cryptic speciation occur (Fontaneto et al. 2009; García-Morales and Elías-Gutiérrez 2013) and, in this sense, the *Brachionus plicatilis* complex represents are good example and one of the best studied groups. The *Brachionus plicatilis* species complex is composed of three major morphotypes: the large (L), the small-medium (SM) and the small size (SS) types (Ciros-Pérez et al. 2001). The species currently described are:

1. *B. asplanchnoidis* Charin, 1947 (L)

2. *B. plicatilis* sensu stricto Müller, 1786 (L)

3. *B. manjavacas* Fontaneto et al., 2007 (L)

4. *B. ibericus* Ciros-Pérez et al., 2001 (SM)

5. *B. koreanus* Hwang et al., 2013 (SM)

6. *B. rotundiformis* Tschugunoff, 1921 (SS)

However, Mills et al. (2017) provided a conservative estimate of nine additional species within the *B. plicatilis* complex, with 11 more likely when applying the automatic barcode gap discovery method to the COI gene.

In the present work, *Brachionus paranguensis* sp. nov., collected in the hypersaline and highly alkaline volcanic maar Rincón de Parangueo, Guanajuato, Mexico (Cerca et al. 2014; Rocha-Treviño 2015) is described. It represents the unofficially known and undescribed *B.* ‘Nevada’ of the L4 clade (Gómez et al. 2002; Mills et al. 2017). The formal description of *Brachionus paranguensis* sp. nov. includes *B.* ‘Nevada’ which does not represent a formal taxonomic identity, and L4 could therefore be officially named *B. paranguensis* sp. nov.

To support the identity of the new species an integrated approach using DNA taxonomy, ecology and morphology was applied. The DNA taxonomy was based on two genes, the mitochondrial gene COI (cytochrome c oxidase I), sometimes referred to as the DNA barcoding gene, and the nuclear ribosomal ITS1 (internal transcribed spacer I). Sequences of *B. paranguensis* sp. nov. were compared with published sequences belonging to the L group of the *B. plicatilis* species complex and phylogenetic analyses were performed to infer the relationship among sequences.

In order to provide further evidence to support the recognition of *B. paranguensis* sp. nov., a morphological description of parthenogenetic females and resting eggs is provided, as well as lifespan analysis. Furthermore, a formal description of the species is given along with potential diagnostic features specific to *B. paranguensis* sp. nov. that could discriminate this species from other members of the L group.

## Material and methods

Water samples were collected with a zooplankton net of 50 μm from the three water bodies located in the Rincón de Parangueo volcano, Guanajuato, Mexico (WGS84 coordinates 20°25'46"N; 101°14'48"W, altitude 1686 m above the sea level). Samples used for taxonomic identification were fixed with 4% formalin, while those intended for DNA analysis were fixed with 96% ethanol. An additional *in vivo* sample was kept for culturing under laboratory conditions. This latter sample was kept in a cooler at 4 °C for no more than 4 hours until cultures were set up in the laboratory.

Physical and chemical parameters were measured in situ using the Yellow Spring Instruments Model 556 MPS probe. Environmental variables included: pH, temperature, dissolved oxygen (DO), conductivity and salinity [calculated with the conductivity to salinity conversion table by Bodelón et al. (1994)].

### DNA taxonomy

From field samples ten specimens were selected for DNA analysis. DNA was extracted from each individual using a mixture of proteinase K with lysis buffer for invertebrates and digested overnight at 56 °C. Genomic DNA was subsequently extracted using the glass fiber membrane method in 2 μm Pall plates (Hajibabaei et al. 2005). Both COI and ITS1 were amplified from the same specimens. For COI the PCR (polymerase chain reaction) reactions were prepared using 6.25 μl of 10% trehalose, 1.63 μl of deionized water, 1.25 μl of 10X Buffer, 0.625 μl of MgCl_2_ (50 mM), 0.31 μl of both primers (0.01 mM), 0.0625 μl of dNTPs (10 mM), 0.0625 μl of Taq polymerase (Invitrogen) and 2 μl of DNA template. The universal COI primers for animals, LCO1490 and HCO2198 (Folmer et al. 1994) were used. The PCR program used included an initial polymerase activation step at 94 °C for 1 min, five cycles of denaturation at 94 °C for 40 s, annealing at 45 °C for 40 s and an extension at 72 °C for 1 min, followed by 35 cycles of 94 °C during 40 s, 51 °C for 40 s and 72 °C for 1 min, with a final extension at 72 °C for 5 min.

For ITS1 the PCR reactions were prepared using 8.85 μl of deionized water, 1.5 μl of 10X Buffer, 1 μl of BSA, 0.6 of μl MgCl2 (50mM), 0.3 μl of both primers (20 μM), 0.3 μl of dNTPs (10 mM) and 0.15 μl Taq polymerase (Platinum). The ITS1 primers used were III (5’-CACACCGCCCGTCGCTACTACCGATTG-3’) and VIII (5’-GTGCGTTCGAAGTGTCGATGATCAA-3’) of Palumbi (1996). The PCR program used included an initial step at 95 °C for 5 min, 36 cycles of 95 °C for 50 s, 54 °C for 50 s, 72 °C for 1 min and one cycle at 72 °C for 5 min.

All amplified products were screened with an agarose E-gel (Invitrogen) and pictures of the positives were taken. The products were labeled with the BigDye Terminator v. 3.1 (Applied Biosystems, Inc.) and sequenced bidirectionally. The DNA sequencing process was conducted at the James Hutton Institute of Dundee (Scotland, UK). All sequences were quality checked with Geneious 4.8 (http://www.geneious.com, Kearse et al. 2012) and with the quality tools provided by the Barcode of Life Database (BOLD, www.boldsystems.org) (Ratnasingham and Hebert 2007). Alignment of COI and ITS1 sequences was performed with Topali v2.5 (Milne et al. 2008) using a F84+Gamma model. All new COI sequences after this work were uploaded in BOLD within the Dataset DS-BPAR01 under the accession numbers BPMX010-19 to BPMX017-19 and GenBank (www.ncbi.nlm.nih.gov) accession numbers MK434153–MK435160. All new ITS1 sequences were deposited in BOLD with accession numbers from BPMX001-18 to BPMX008-18 and GenBank MH708047–MH708053.

Both COI and ITS1 sequences of *B. paranguensis* sp. nov. were aligned against all *B. plicatilis* complex L clade COI and ITS1 sequences available in the NCBI (National Center for Biotechnology Information) public database. Sequences were trimmed to a sequence length of 550 bp for COI and to 350 bp for ITS1, and those that did not cluster with the members of the L group were discarded. A total of 665 COI and 148 ITS1 sequences belonging to the whole L group of the *B. plicatilis* complex were retained for this study. One COI sequence, KU299077 (Mills et al. 2017), was not used for analyses as it was poor with too much missing information. Likewise, two ITS1 sequences, AY772137 and AY772159 (Suatoni et al. 2006) identified as *B. plicatilis* s.s. and *B.* ‘Nevada’ respectively, were excluded from the analyses. A BLAST search in the NCBI database showed the former sequence to be 100% similar to another sequence, KU299536, belonging to the SM group (Mills et al. 2017); while AY772159 was placed outside the four L groups presenting a 22% genetic distance from the other *B.* ‘Nevada’ clones.

Sequence alignments were reduced to haplotypes by collapsing all identical sequences, using the online toolbox users-birc.au.dk/biopv/php/fabox/dnacollapser.php# (Villesen 2007). In order to infer the relationship among *B. paranguensis* sp. nov. and the other species of the L group of the *B. plicatilis* complex, a maximum likelihood tree (ML) was constructed for the COI and ITS1 datasets with PhyML 3.0 (Guindon and Gascuel 2003). Two sequences of the congeneric species *Brachionus rotundiformis*, AF387287 and AF387239 for COI and ITS1 respectively, were used as outgroups as this species represents the closest one to the L group (Mills et al. 2017). The best model was identified as TN93+G for COI, and GTR+G for ITS1 dataset. In order to set an appropriate threshold value able to define species belonging to the L group of the *Brachionus* complex, mean uncorrected p-distances were calculated within and between members of the group, with Mega 7.0.21 (Kumar et al. 2016).

### Morphology

A Light Microscope (LM) (Nikon Eclipse Ni) and a Scanning Electron Microscope (SEM) were used to analyze the morphology of *B. paranguensis* sp. nov. females, males, trophi, and eggs. Specifically, the presence of gastric glands, the shape of the dorsal sinus and the anterior spines of the female were analyzed by LM. Whereas, the lorica surface structure, the presence of lateral antennae, the foot aperture in the female, the male, the three different type of eggs, and the trophi, were analyzed by SEM. All samples were taken directly from the same field samples as the material for DNA analyses with the exception of the males, which were taken from laboratory cultures.

### Preparation of organisms for SEM

One hundred females, fifty males, twenty parthenogenetic eggs, twenty mictic eggs and twenty unfertilized eggs were extracted from the field samples and fixed in 4% formalin. Specimens were dehydrated using a graded ethanol series, starting with 60% and finishing with 100% ethanol; after which critical drying point was performed. Samples were then mounted in a SEM stub (1 cm high x 1.2 cm in diameter) and covered with gold. For the preparation of trophi a sample of 20 female organisms was used. Trophi were removed according to the methodology of Segers et al. (1993). The organisms and trophi were mounted on glass slides and observed under SEM JEOL 5900 LV; pictures were taken according to Silva-Briano et al. (2015).

### Morphometric analysis

**Figure 1.**
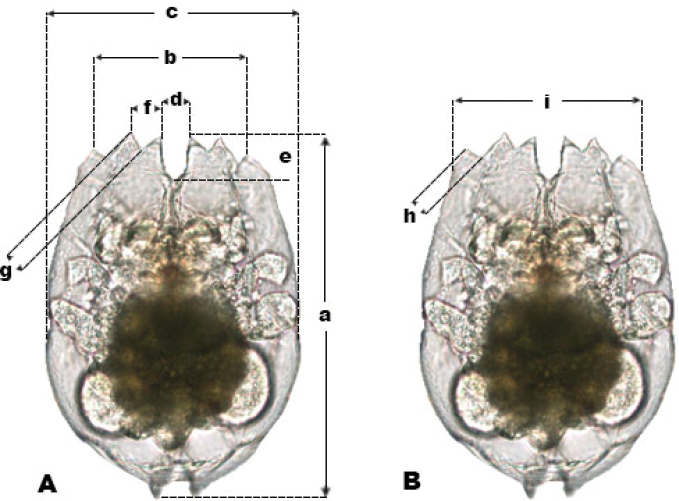
Different types of measurements. Dorsal view with the strokes of how anterior spines were measured according to **A**Fu et al. (1991) and **B**Ciros-Pérez et al. (2001).

New-born females were isolated from cultures and placed in a well of a 24-well polystyrene plate for 48 hours. In order to compare morphometric data of *B. paranguensis* sp. nov. against the other members of the L group, females were fixed with 4 % formaldehyde and placed all in the same position under the optical microscope where twenty digital pictures were taken. Organisms were placed and measured according to Fu et al. (1991) and Ciros-Pérez et al. (2001), see Figure 1, using the program provided by the microscope Nikon Eclipse Ni with digital camera D5-Fi2.

### Ecological analysis

**Culture conditions**

Cultures were set up from several wild individuals collected in the volcanic maar Rincón de Parangueo, using the same salinity recorded in situ, 25 g L^-1^. Cultures were maintained in a bioclimatic chamber with a photoperiod of 16: 8 light: dark, a temperature of 25 °C and a medium with 25 g L^-1^ of RED-SEA SALT (Ca 410 ppm, Mg 1230 ppm, and AlK / KH Meq / l / 7.7 dKH) dissolved in distilled water (pH = 8.5). Rotifers were fed with 10^6^ cells/ml concentration of the algae *Nannochloropsis oculata*.

**Lifespan of parthenogenetic female and males**

Lifespan analysis of males and females was conducted under laboratory conditions in order to provide ecological information of the species. In 1 mL of medium, 10 neonates of parthenogenetic females were isolated in individual wells of a 24-well polystyrene plate until they died. Every 24 hours neonates of each female were collected from the wells. Rotifers were fed with 10^6^ cells/ml of *Nannochloropsis oculata* and placed into a new well with fresh medium in a total volume of 1 ml, every day during the experiment. The mean lifespan, the number of eggs per rotifer, and the maximum intrinsic growth rate were calculated for parthenogenetic females according to Krebs (1985) and Begon et al. (1996). Mean lifespan was calculated from twenty males collected within six hours of hatching.

## Results

### DNA taxonomy

**Figure 2.**
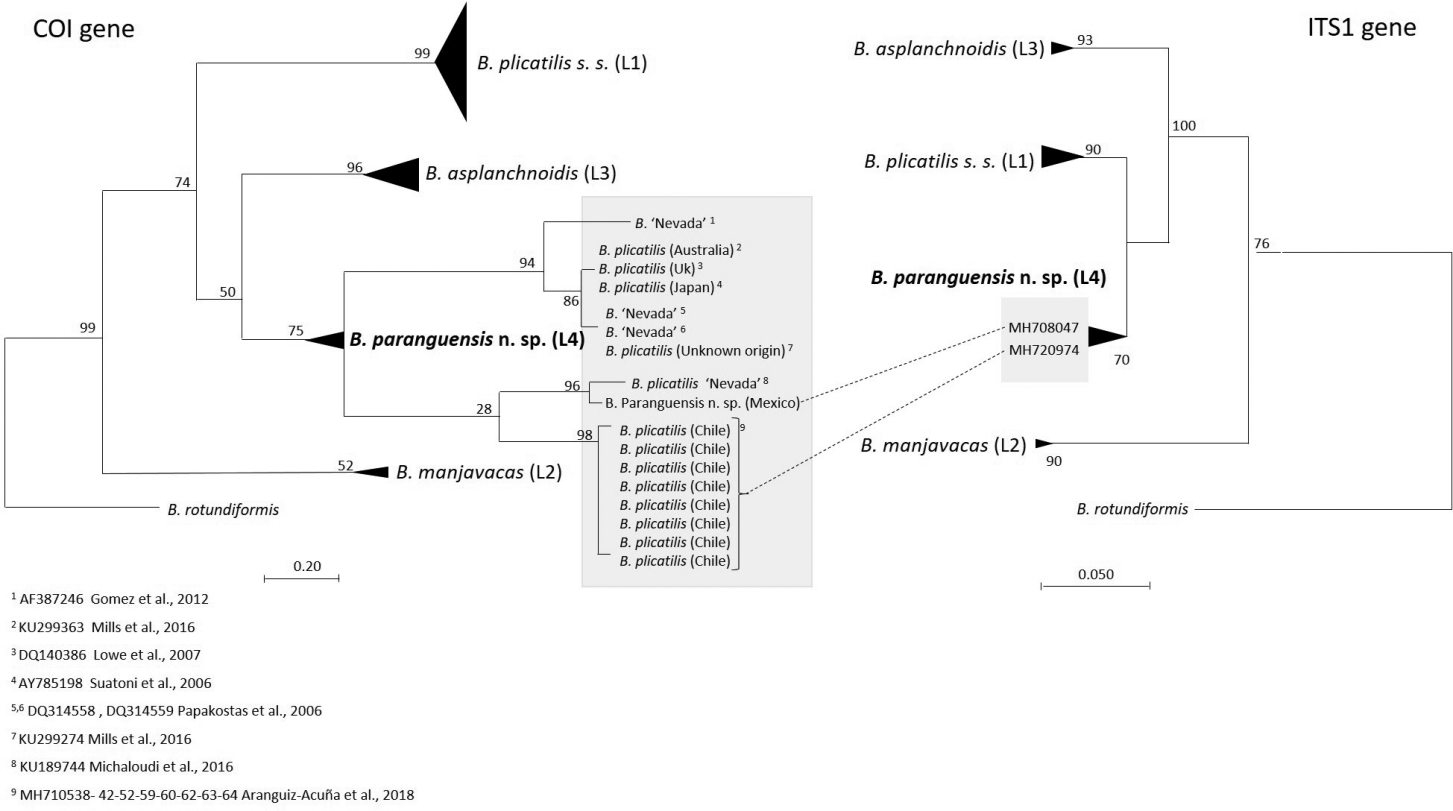
Comparison of COI and ITS1 LM trees focusing on the L4 group. COI and ITS1 Maximum Likelihood trees representing the four L groups of the *Brachionus plicatilis* complex and one outgroup species *Brachionus rotundiformis*. Focus is placed upon *B. paranguensis* sp. nov. L4 clade (grey shading) where details of the origin of each haplotype are given; all the other species are collapsed. Dotted lines between haplotypes indicate COI and ITS1 sequences that were sourced from the same individuals. Numbers at nodes represent support values (bootstrap = 1000). A list of references for each haplotype within the L4 group is provided.

From the 10 processed individuals of *Brachionus paranguensis* sp. nov., eight sequences were obtained for each marker, COI and ITS1, from the same individuals. For both markers, sequences belonged to a single haplotype (MK434153 and MH708047, respectively). The COI alignment that included 665 sequences belonging to members of the L group, collapsed into 146 haplotypes; while ITS1 alignment contained 148 sequences and collapsed into 12 haplotypes. The ML trees for both COI and ITS1, formed four well-defined clusters representing the four L clades of the *B. plicatilis* complex (Mills et al. 2017) (Suppl. material 1: Figs S1, S2). For COI, mean uncorrected genetic distances within groups ranged from 2.8% to 8.5% (mean = 4%, media = 4%), while distances between groups ranged from 15% to 19.8% (mean = 16%, median = 16%) (Table 1). *Brachionus paranguensis* sp. nov. haplotype was included in the L4 clade unofficially known as ‘Nevada’. The L4 clade presented 17 haplotypes; besides *B. paranguensis* sp. nov., these were represented by clones of *B. plicatilis* collected from Chile (Aránguiz-Acuña et al. 2018), UK (Lowe et al. 2007), Australia (Mills et al. 2017), Japan (Suatoni et al. 2006), and clonal cultures belonging to the undescribed *B.* ‘Nevada’ (Gómez et al. 2002; Papakostas et al. 2006; Michaloudi et al. 2017) (Fig. 2). The mean uncorrected p-distance within the L4 clade was 8.5%, ranging from 0 to 15%. For ITS1, mean uncorrected genetic distances within groups ranged from 0.001% to 0.7% (mean = 0.4%, median = 0.3%), while distances between groups ranged from 2.8% to 9.3% (mean = 6.6%, median = 6%) (Table 2). Congruently with the results obtained for COI, *B. paranguensis* sp. nov. haplotype was included in the L4 clade, together with another haplotype belonging to a clone of *B. plicatilis* collected from Chile (Aránguiz-Acuña et al. 2018) (Fig. 2). The haplotype was sourced from the same individual as one of the haplotypes present in the L4 clade of the COI ML tree. No variability in the ITS1 fragment was found between *B. paranguensis* n. sp and three clones named *B.* ‘Nevada’ (LC339820, FR729715 and AF387207) available in GenBank. The mean uncorrected p-distance within the L4 clade was 0.001%.

**Table 1.** COI genetic distances among and within the members of the L group. COI uncorrected p-distances generated within and among members of the L group. In black are within distances, while lower left are between mean p-distances.

	L1	L2	L3	L4
L1	3.6%			
L2	19.8%	2.8%		
L3	18%	19.7%	4%	
L4	18.9%	17.7%	15%	8.5%

**Table 2.** ITS1 genetic distances among and within the members of the L group. ITS1 uncorrected p-distances generated within and among members of the L group. In black are within distances, while lower left are between mean p-distances. Within distance for L2 has N/A as only one haplotype is present.

	L1	L2	L3	L4
L1	0.7%			
L2	8.9%	N/A		
L3	6.3%	9.3%	0.6%	
L4	2.8%	7.6%	5%	0.001%

## Taxonomy

### Class Eurotatoria De Ridder, 1957

#### Subclass Monogononta Plate, 1889

**Superorder Pseudotrocha Kutikova, 1970**

**Order Ploima Hudson & Gosse, 1886**

**Family Brachionidae Ehrenberg, 1838**

##### Brachionus paranguensis sp. nov.

Taxon classificationAnimaliaPloimaBrachionidae

6560750D-1719-5E1F-B5E5-8FC2202E1B63

http://zoobank.org/2BE62C89-4A13-40EE-B395-08E8B13B3971

Figs 4A–H , 5A–C , 6A–F

###### Type locality.

**Figure 3.**
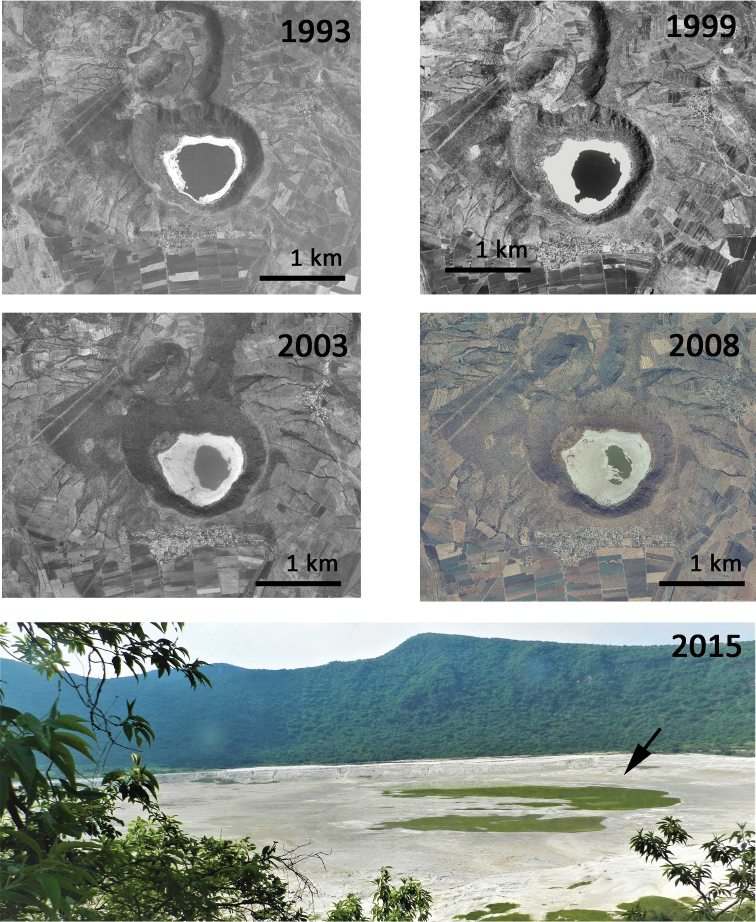
Map of the studied area. Sequence of pictures of the volcanic maar Rincón de Parangueo from 1993 to 2015, showing how the lake gradually desiccated. The arrow indicates the water pond where *B. paranguensis* sp. nov., was found (digital pictures downloaded from INEGI 2019).

The volcano Rincón de Parangueo, Guanajuato, Mexico, has two or three water bodies inside the crater, depending on the season, and *B. paranguensis* sp. nov. was present in one of them, 20°25'46"N; 101°14'48"W, at the altitude of 1686 m above sea level (Fig. 3).

###### Material examined.

In order to confirm the identity of *B. paranguensis* sp. nov., hundreds of individuals from field and culture samples representing all stages (amictic females, males, resting eggs, parthenogenetic eggs, and unfertilized sexual eggs), and around 30 trophi, were examined by LM and SEM. Specifically, hundreds of females were used to take morphometric measurements, and dozens of males were observed to analyze their morphological features. Moreover, diapausing eggs, parthenogenetic eggs and unfertilized eggs were examined and pictures of each egg were taken. Finally, approximately 30 trophi were analyzed in order to compare their morphological features with those of the other eggs belonging to the L group.

***Holotype.*** A parthenogenetic female mounted on a slide with a mix of formaldehyde-glycerol sealed with DePex medium, deposited in the Zooplankton Reference Collection of El Colegio de la Frontera Sur with accession number ECO-CH-Z-10331. ***Paratypes.*** Two slides with a parthenogenetic female, deposited in the Zooplankton Reference Collection of El Colegio de la Frontera Sur with accession numbers ECO-CH-Z-10332, 10333.

###### Differential diagnosis.

***Parthenogenetic female***: No clear morphological differences were observed between *B. paranguensis* sp. nov. and the other species belonging to the L group of the *B. plicatilis* complex with respect to the anterior dorsal spine, the U-shape sinus, the orange peel like surface of the lorica, and the presence of gastric glands. An exception is *B. asplanchnoidis*, whose lorica presents an elongated and wider shape, and the antero-ventral U-shape sinus is wider compared to that of the other members.

*Trophi*: In *B. paranguensis* sp. nov. satellites are robust and there are sharp projections in the inner upper margin; basifenestras with similar size and shape, ramus with two posterior projections, the left one smaller and thinner than the right one. In *B. plicatilis* s.s. the shape of the satellites is triangular with no projection; ramus with two posterior projections, the left one bigger and wider than the right one; basifenestras asymmetrical with different sizes. In *B. manjavacas* the shape of the satellites is triangular and sharper compared to *B. plicatilis* s.s.; ramus with two posterior projections, the left one bigger and wider than the right one; basifenestras with same shape but different size, the left one bigger than the right one. In *B. asplanchnoidis* satellites are robust and the projections in the inner upper margin is rounded; ramus with two posterior projections, the left one smaller and thinner than the right one, the right one with cylindrical shape; basifenestras with different shape and size, the left one smaller than the right one.

*Resting eggs*: Oval shape in *B. paranguensis* sp. nov., *B. plicatilis* s.s. and *B. manjavacas*, although the two latter species also present small holes on the surface of the eggs (Ciros-Pérez et al. 2001; Guerrero-Jiménez et al. in prep.).

*Ecology: B. paranguensis* sp. nov. grows preferentially in salinities higher than 25 g L^-1^. *B. plicatilis* s.s. grows better in salinities from 5 to 15 g L^-1^ (Yin and Zhao 2008), while *B. manjavacas* is usually cultured in a salinity of 12 g L^-1^. Finally, *B. asplanchnoidis* can be observed in salinities from 8 to 44 g L^-1^, although Michaloudi et al. (2017) for their study cultured the species in a salinity of 16 g L-1.

###### Description.

**Figure 4.**
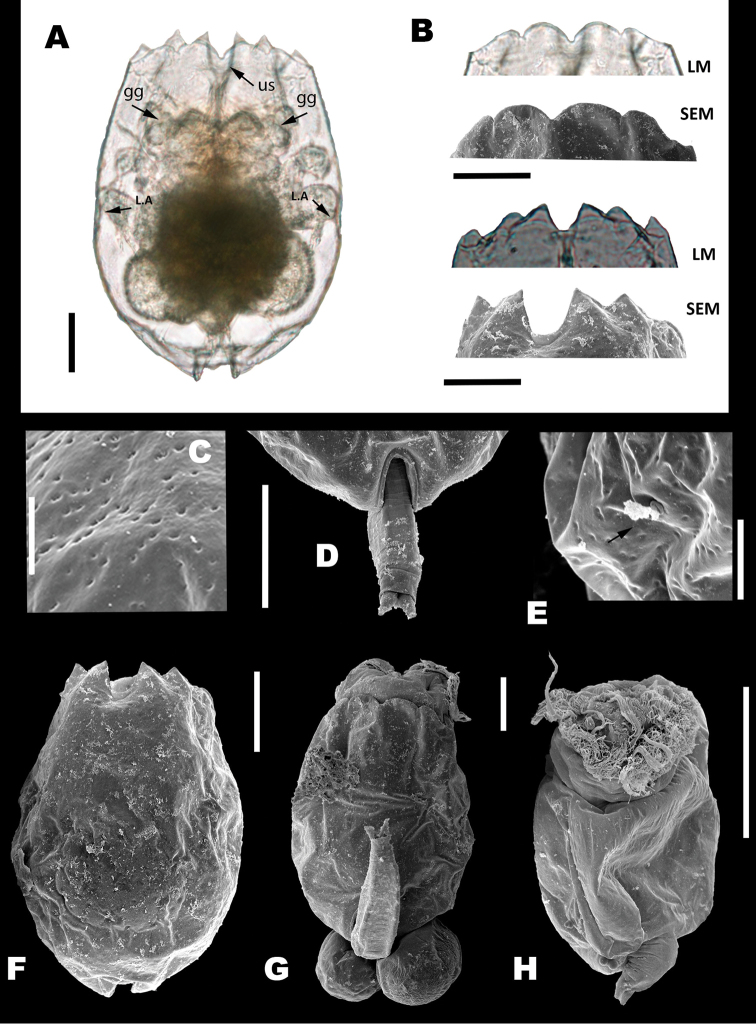
Taxonomic features of *B. paranguensis* sp. nov. **A** parthenogenetic female with lateral antennae (LA), gastric glands (gg), an U-shape sinus (us) **B** anterior dorsal and ventral spines taken by LM and SEM**C** lorica with an orange peel like surface **D** foot aperture **E** lateral antenna (see arrow) **F**SEM microphotographs of the female, ventral plate and **G** dorsal plate and **H** male. Scale bars: 50 µm (**A, B, D, F, G, H**), 10 µm (**C, E**). All females from sample collected in June 27, 2015. Males from cultures obtained from females collected in the volcanic maar Rincón de Parangueo.

The population of *B. paranguensis* sp. nov. from Rincón de Parangueo volcano was used to formally describe all individuals belonging to the L4 group of the *B. plicatilis* complex. Parthenogenetic females (Fig. 4F, G) had a soft lorica with an orange peel like surface (Fig. 4C), two gastric glands (gg) (see arrows, Fig. 4A) and two lateral antennae (LA) in the middle of the body (see arrows, Fig. 4A, E). Anterior dorsal margin with six spines, three on each side of a U-shape sinus like the other members of the L group. All spines are triangular (Fig. 4A, B). Anterior ventral margin with bilateral symmetry, four well-defined lobes with a medial sinus, both internal lobes were more pronounced than the external lobes (Fig. 4A, B). Foot aperture well defined (Fig. 4D). Adult females had a length of 216.97 ± SD 13.78 μm (*N* = 20), width 159.93 ± SD 10.93 μm (*N* = 20), and 126.21 ± SD 7.7 μm of head aperture, dorsal view (*N* = 20), see Table 3.

***Male*** (Fig. 4H): Twenty individuals were analyzed. Anterior ventral margin had two lobes with a medial sinus and five to six pellets, the life cycle without food was of four days and its length was 78.48 ± SD 2.8 μm and width 72.7 ± SD 2.3 μm.

**Table 3.** Measurements of all species belonging to the L group. Measurements of adult females between all species belonging to the L group, according to Fu et al. (1991) and Ciros-Pérez et al. (2001). All measurements not belonging to *B. paranguensis* sp. nov. were taken from Michaloudi et al. (2017).

**Group L**	**Measurements**								
	**a**	**b**	**c**	**d**	**e**	**f**	**g**	**h**	**i**
*B. plicatilis* s. s. (L1)	283.63 ± 6.38	122. 36 ± 1.72	203.62 ± 5.49		27.33 ± 0.42		13.74 ± 0.31		132.44 ± 2.13
*B. manjavacas* (L2)	256.28 ± 3.9	109.37 ± 1.75	177.67 ± 2.62		21.74 ± 0.52		10.04 ± 0.28		125.09 ± 1.91
*B. asplanchnoidis* (L3)	295.12 ± 8.1	126.12 ± 1.85	204.58 ± 4.8		26.64 ± 0.75		14.42 ± 0.45		130.88 ± 2.12
*B. paranguensis* sp. nov. (L4) *N* = 20	216.97 ± 13.78	106.43 ± 8.13	159.93 ± 10.93	25.43 ± 2.88	23.82 ± 2.23	20.13 ± 2.06	9.75 ± 0.81	12.93 ± 1.09	126.21 ± 7.72

**Figure 5.**
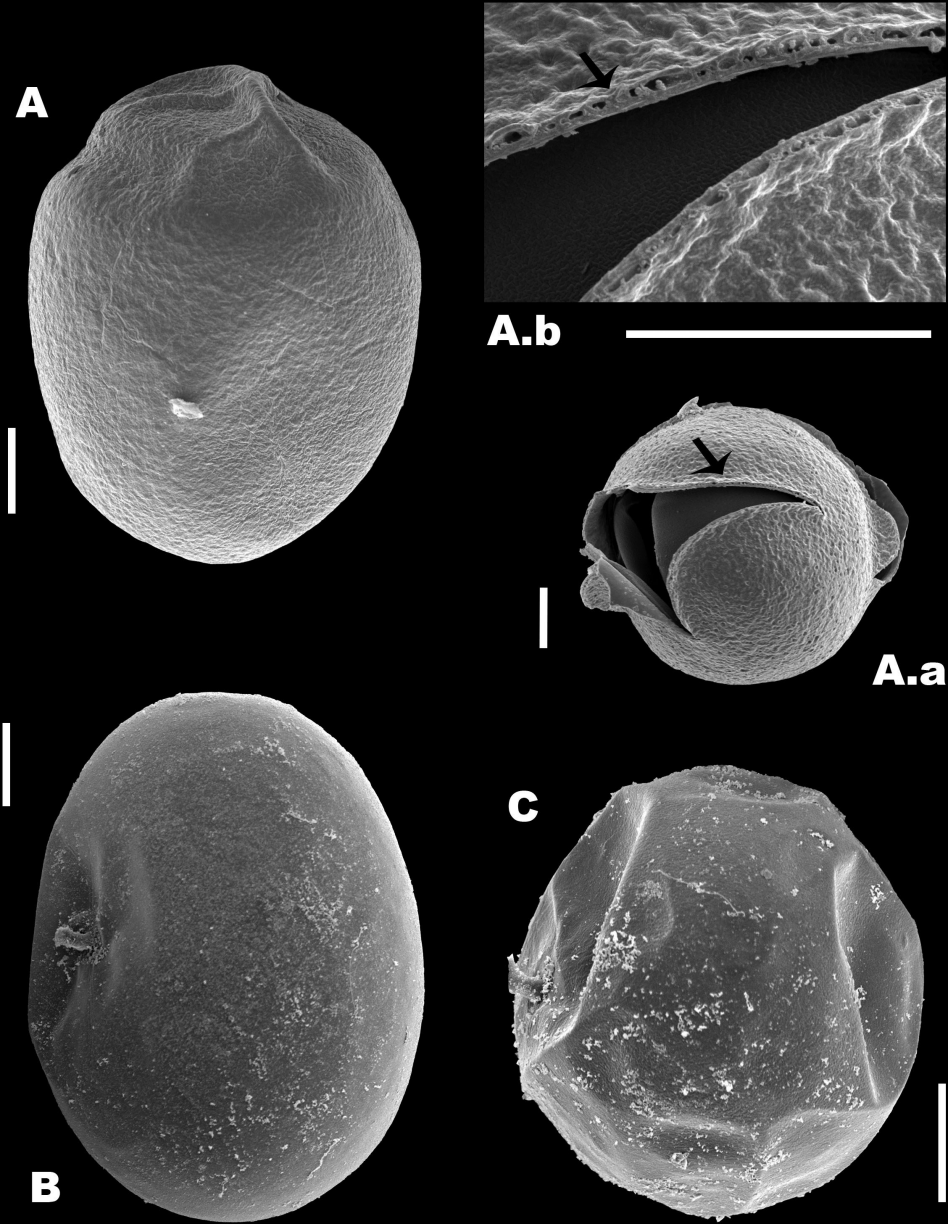
Types of eggs in *B. paranguensis* sp. nov. SEM micrographs of the different types of eggs of *B. paranguensis* sp. nov. **A** resting egg from field samples and its special ornamentation and zoom of its membrane, see arrows (A.a, A.b) **B** parthenogenetic egg and **C** unfertilized sexual egg, both from cultured samples. Scale bars: 20 µm

*Egg types*: The outer membrane of the resting eggs presented a slightly rough ornamentation (Fig. 5A.a, A.b) and the length was 112.6 ± SD 4.8 μm and 91.2 ± SD 4.7 μm width (*N* = 7), see Figure 5A. The parthenogenetic eggs had a smooth surface and was 127.4 ± 9.5 long and 110.7 ± SD 6.1 μm wide (*N* = 9), see Figure 5B. Finally, the unfertilized sexual eggs that produced males were 73.03 ± SD 3.2 μm long and 59.8 ± SD 2.18 μm wide (*N* = 9), see Figure 5C.

**Figure 6.**
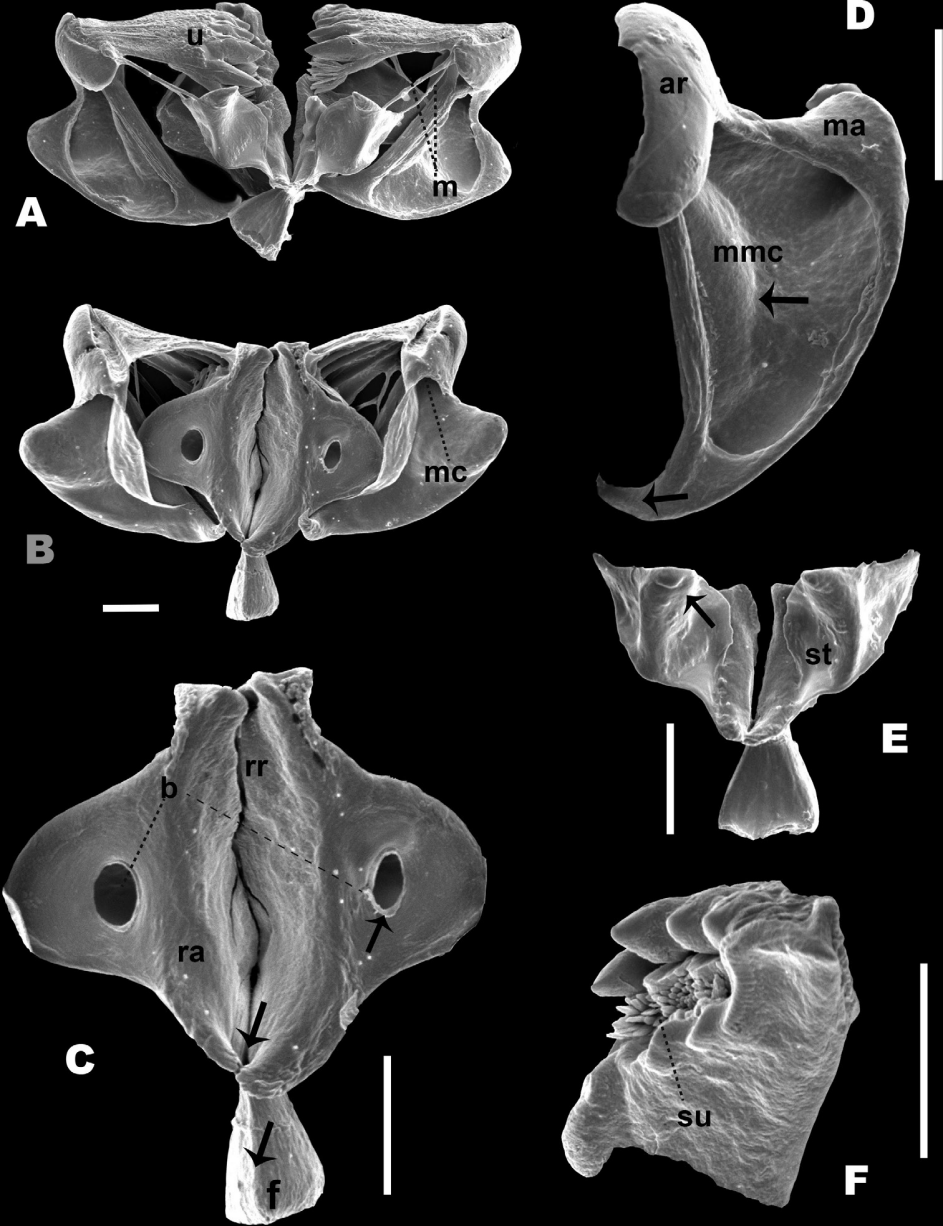
Trophi components of *B. paranguensis* sp. nov. SEM pictures of the trophi components of *B. paranguensis* sp. nov., collected in June 27, 2015 **A** ventral view **B** dorsal view **C** rami and fulcrum **D** manubrium **E** satellites **F** unco and sub uncus ar: articulation of manubrium, m: membrane; ma: manubrium with posterior claw, see arrow (**D**); mmc: manubrium middle crest, see arrow (**D**); rr: reinforced ridge; st: satellites, see arrow (**E**); su: sub uncus; u: uncus.: b: basifenestras, see arrow (**C**); f: fulcrum, see arrow (**C**); mc: manubrium cavities; ra: rami, and projections of the posterior portion of the rami, see arrow (**C**)). Scale bars: 10 µm.

*Trophi*: Malleate type with all the characters of the genus (Fig. 6A, B). Manubria were triangular in shape with sharp claw-shaped tips at their distal end (Fig. 6D). The junction that holds the manubrium with the uncus was wide in both dorsal and ventral view (Fig. 6A, B). Unci presented four teeth decreasing in size from the ventral one and the subuncus is present underneath each tooth (Fig. 6F). The membrane that joins the uncus with the satellites was thick and the protrusion that innervates them was also clearly denoted on the back of the uncus (see Fig. 6A). The satellites were robust, irregularly shaped but arranged symmetrically, also sharp projections were observed on the anterior section of the satellites with the tip pointing inwards towards the central axis of the trophi (Fig. 6E). Ramus asymmetrical with two posterior projections, the left one smaller and thinner than the right one and basifenestras with similar shape and size (Fig. 6C). Fulcrum short, with triangular shape (Fig. 6C, see arrow and F).

###### Etymology.

The specific name refers to the type locality, the volcanic maar Rincón de Parangueo.

###### Distribution-habitat.

According to the DNA sequences available in NCBI, *B. paranguensis* sp. nov. individuals were reported in Mexico, USA, Chile, Europe, Australia, and Japan. The habitat of *B. paranguensis* n. sp is represented by high salinity environments (>25 g L^-1^). Physical and chemical parameters of the maar where specimens were collected: Temperature= 28 °C; Conductivity= 43.2 mS/cm^-1^; Dissolved Oxygen = 5.5 mg L^-1^; pH= 11.07.

###### Ecology.

Under laboratory conditions (25 °C, 25 g L^-1^ salinity, 10^6^ cells/ml of *Nannochloropsis oculata* provided as food) amictic females had a maximum lifespan of 10.8 days. Eggs per rotifer were 15.4 ± SD 5.8, and the maximum intrinsic growth rate was 0.49 (*N* = 10).

## Discussion

### DNA taxonomy

Due to the morphological stasis of the external characters that characterizes the *B. plicatilis* species complex (Gómez et al. 2002; Campillo et al. 2005; Mills et al. 2017) the use of genetic analysis represented a fundamental aid to help unravel the cryptic species diversity within this group, in combination with other diagnostic characters. Additionally, its discrimination power among species is reliable, as was demonstrated in previous studies (García-Morales and Elías-Gutiérrez 2013; Mills et al. 2017).

The current taxonomy of the L group has not been resolved yet, leading to an underestimation of the true diversity present within the complex (Gómez et al. 2002; Mills et al. 2017). An enormous confusion is currently present due to sequencing only resting eggs from localities not accurately described, for example, Little Fish Pond (Gómez et al. 2002; Suatoni et al. 2006, Mills et al. 2017) or even China (Mills et al. 2017). Moreover, some members of the group lack a formal taxonomic description, therefore unofficial names are used to identify taxa, which may create problems when communicating about the group or comparing results between studies (Serra and Fontaneto 2017). Thus, a common effort is required to provide unambiguous names thet al.ow a link between the name and the taxonomic identity (Segers et al. 2012). In the present study, DNA taxonomy through the use of the two markers COI and ITS1, represented an important tool that helped confer an identity to the yet undescribed *B.* ‘Nevada’, named here as *B. paranguensis* sp. nov.

Phylogenetic analyses using both markers clustered *B. paranguensis* sp. nov. and *B.* ‘Nevada’ together as a single species, although a higher genetic variability in COI was observed within haplotypes (8.5%, ranging from 0 to 15%). Sequences of *B. paranguensis* sp. nov. revealed no variability in ITS1 with three clones of *B.* ‘Nevada’ available in GenBank, representing therefore a single haplotype, with only a single base-pair difference with the other haplotype belonging to a clone of *B. plicatilis* collected from Chile, included in the L4 clade.

Previous genetic analysis with the COI gene reported high genetic divergences within the *B. plicatilis* species complex up to about 20% (Fontaneto 2014), and a similar maximum intraspecific divergence of 15% was found in this study within haplotypes included in L4. This value represents a much higher threshold than the 0.03 cut off commonly used in animal studies for delimiting species using COI (Hebert et al. 2003). Studies conducted on other animal groups, such as the springtail *Friesea grisea* (Torricelli et al. 2010) or the copepod *Tigriopus californicus* (Edmands 2001), also reported the presence of higher genetic divergences in COI sequences, demonstrating the impracticability of applying a 0.03 to all animal taxa. The mitonuclear discordance between COI and ITS1 in rotifers has already been observed in previous studies (Papakostas et al. 2016) as the mitochondrial gene COI was shown to evolve more rapidly than the nuclear ribosomal internal transcribed spacer ITS1 (Tang et al. 2012). The high mutational rate of COI might explain the high genetic variation found for this gene within *Brachionus* species and, therefore, the 8.5% mean genetic divergence existing within haplotypes in L4. To avoid the over-splitting of *Brachionus* species, which occurs when using COI marker, ITS1 has been suggested as a more reliable predictor of the species in the *B. plicatilis* complex (Papakostas et al. 2016; Mills et al. 2017). In view of the above, it is possible to affirm that, according to DNA taxonomy analysis, *B. paranguensis* n. sp and *B.* ‘Nevada’ represent the same taxonomic identity.

### Morphology

Morphological differentiation among parthenogenetic females belonging to the L group species of the *B. plicatilis* complex is poor. The high variability in size and the lack of differentiation between the anterior dorsal and ventral margins, already reported for *B. asplanchnoidis* by Michaloudi et al. (2017), may also occur in other species. However, it is worth mentioning that Michaloudi et al. (2017, 2018) observed that the ventral margin represented a helpful diagnostic character in females of *B. asplanchnoidis* and *B. calyciflorus*. Other morphological similarities were found among the members of the L group, i.e., the presence of gastric glands, the lorica with an orange peel like surface, and the U-shape sinus. Therefore, so far, no reliable morphological features that could allow distinguishing among species have been observed in females. However, for *B. paranguensis* sp. nov. some differences were observed in the trophi, in the resting eggs ornamentation, and in the salinity preference.

Trophi are indeed relatively consistent features in rotifers (Segers et al. 1993; Fontaneto and Melone 2005) and are already used as valid diagnostic features for identification in some bdelloids (Melone and Fontaneto 2005). Although there is no evidence of species specificity in the ultrastructure of the trophi, small differences were observed in some components, especially in the satellites, among the species of the L group. These could be used for a preliminary identification of the species as an alternative to DNA taxonomy.

The study of resting eggs morphology could represent another alternative for species differentiation. Indeed, in rotifers, evidence of species specificity in resting eggs was already documented by Nipkow (1961), Beauchamp (1952), Bogoslovsky (1967), and Gilbert and Wurdak (1978). When comparing the ultrastructure of resting eggs in the members of the L group, differences were observed. *Brachionus plicatilis* s.s. eggs have a quite smooth surface (Ciros-Pérez et al. 2001), while in *B. manjavacas* the surface presents wrinkles (Guerrero-Jiménez et. al., in prep). Moreover, small holes were observed on the surface of the eggs belonging to these two species, unlike *B. paranguensis* sp. nov. Although the analyses of *B. asplanchnoidis* eggs is not yet available, so far data seem to support the presence of species specificity among the members of the L group of the *B. plicatilis* complex.

### Ecology

Differences in salinity preference among the species belonging to the L group may represent another factor thet al.ows discriminating between species. Nevertheless, no studies have been carried out on specific salinity preferences of the L group species. Available data reveal that in *B. plicatilis* s.s, a peak in the population growth rate was obtained at a salinity of 10 to 15 g/L^-1^, and then declined when salinity increased (Yin and Zhao 2008). Montero-Pau et al. (2011) presented similar results for *B. manjavacas*, demonstrating that when *B. manjavacas* and *B. plicatilis* s.s. coexist, both in the field and under laboratory conditions, the former species tolerates higher salinity better than *B. plicatilis* s.s. As regards *B. asplanchnoidis*, a study of Papakostas et al. (2013) carried out in Koronia Lake showed a salinity tolerance range of the species of 3.8–8.5 g L^-1^, although Michaloudi et al. (2017) conducted experiments at 16 g/L^-1^ salinity. *Brachionus paranguensis* sp. nov., represents the species better adapted to high salinity concentrations (>25 g L^-1^) as documented by Kostopoulou et al. (2006, 2007, 2009), who cultured individuals of *B.* Nevada at salinities above 30 g L^-1^. Although *B. paranguensis* sp. nov. can be cultured using lower salinities, according to the results obtained in the present study from preliminary tests performed at 15 g/L^-1^ salinity, the lifespan, the number of eggs per rotifer, and the maximum intrinsic growth rate, drastically decreased. These results are also confirmed by Aránguiz-Acuña et al. (2018) who looked at the different response to salinity (from 2.5 to 10 g L^-1^) in two *Brachionus* species, namely *B. quadridentatus* and *B. paranguensis* sp. nov.. While the former species did not survive at the highest salinity, *B. paranguensis* n. sp was positively affected by higher salinity which led to an increased growth rate. This high tolerance to salinity allowed the species to colonize the volcanic maar, representing the only active species found in the water column; resting eggs of two other species, *B. dimidiatus* and *Hexarthra* sp., were also found in the sediment of the water body. Before the 1980s the volcanic maar Rincón de Parangueo was a big lake, with a much lower salinity concentration, until a fracture inside the crater occurred, and the lake started to gradually desiccate (Aranda-Gómez et al. 2013). As the lake dried out, water became hypersaline and highly alkaline as reported by Cerca et al. (2014). Unfortunately, there is no evidence of the presence of *B. paranguensis* sp. nov. in the lake in the past when salinity was much lower, as no studies of the zooplankton community have been previously conducted in this area.

## Conclusion

A formal taxonomic description has been provided for *B. paranguensis* sp. nov. combining DNA taxonomy, morphology and ecology, and results confirmed the identity of the species within the L group.

Based on DNA taxonomy, both COI and ITS1 markers placed *B. paranguensis* sp. nov. within the L4 clade of the *B. plicatilis* complex, confirming that *B. paranguensis* sp. nov. and *B.* ‘Nevada’ represent the same taxonomic identity. Results of genetic variability within the L4 clade were different among markers, with a higher COI intraspecific variability (8.5%) compared to the low 0.001% divergence in the ITS1. High genetic divergence in the COI marker is not unusual and its reliability for predicting species in the *B. plicatilis* complex has already been questioned due to its high mutational rate that leads to an over-splitting of the species. ITS1 has been therefore suggested as a more reliable marker for DNA taxonomy.

Comparison of SEM images among the trophi of *B. paranguensis* sp. nov., *B. plicatilis* s.s., *B. manjavacas*, and *B. asplanchnoidis*, indicate clear differences, including basifenestras, ramus posterior projections and satellites. Resting eggs morphotypes seemed to be species specific, although a comparison with *B. asplanchnoidis* resting eggs is also necessary. A differentiation of parthenogenetic females is only possible between *B. asplanchnoidis* and the other members of the L group, as no clear diagnostic characters are observed between females of the other L clades.

Ecological results on salinity preferences showed that *B. paranguensis* sp. nov. is the species better adapted to hypersaline water bodies. Indeed, results demonstrated that high salinity (>25 g L^-1^) positively affected individuals of *B. paranguensis* sp. nov., while the other members of the L group presented an optimum growth rate at salinities lower than 15–16 g L^-1^.

## Supplementary Material

XML Treatment for Brachionus paranguensis

## Supplementary materials

Supplementary material 1 Figures S1–S2 Data type: molecular data Explanation note: **Figure 1.** COI (TN93+G) Maximum Likelihood tree obtained with PhyML 3.0 of 146 haplotypes belonging to the four L clades of Brachionus plicatilis complex and one outgroup species Brachionus rotundiformis (AF387287). **Figure 2.** ITS1 (GTR+G) Maximum Likelihood tree obtained with PhyML 3.0 of 12 haplotypes belonging to the four L clades of Brachionus plicatilis complex and one outgroup species Brachionus rotundiformis (AF387239). https://binary.pensoft.net/file/346570This dataset is made available under the Open Database License (http://opendatacommons.org/licenses/odbl/1.0/). The Open Database License (ODbL) is a license agreement intended to allow users to freely share, modify, and use this Dataset while maintaining this same freedom for others, provided that the original source and author(s) are credited.Gerardo Guerrero-Jiménez, Patrizia Elena Vannucchi, Marcelo Silva-Briano, Araceli Adabache-Ortiz, Roberto Rico-Martínez, David Roberts, Roy Neilson, Manuel Elías-Gutiérrez
