# Supplementary figures and images for: Brachionus paranguensis sp. nov. (Rotifera, Monogononta), a member of the L group of the Brachionus plicatilis complex
Source: Zookeys. 2019 Oct 14;880:1–23. doi: 10.3897/zookeys.880.28992 (PMC6803355; doi:10.3897/zookeys.880.28992)

0.20      0.40      0.60      0.80      1.0      1.2      1.4

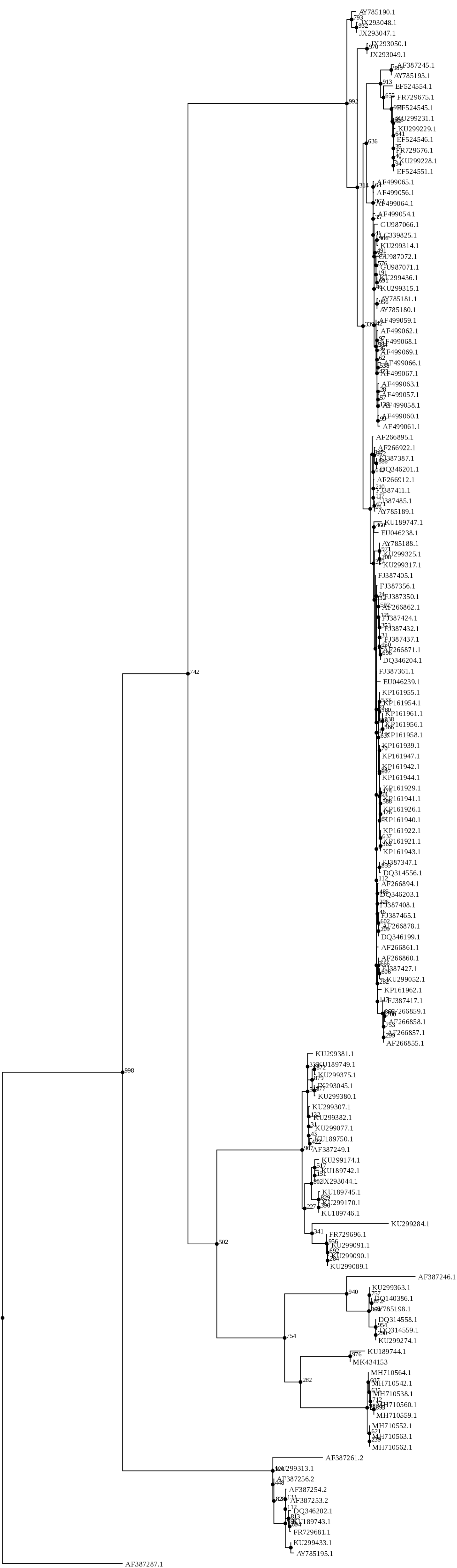

0.050

0.10

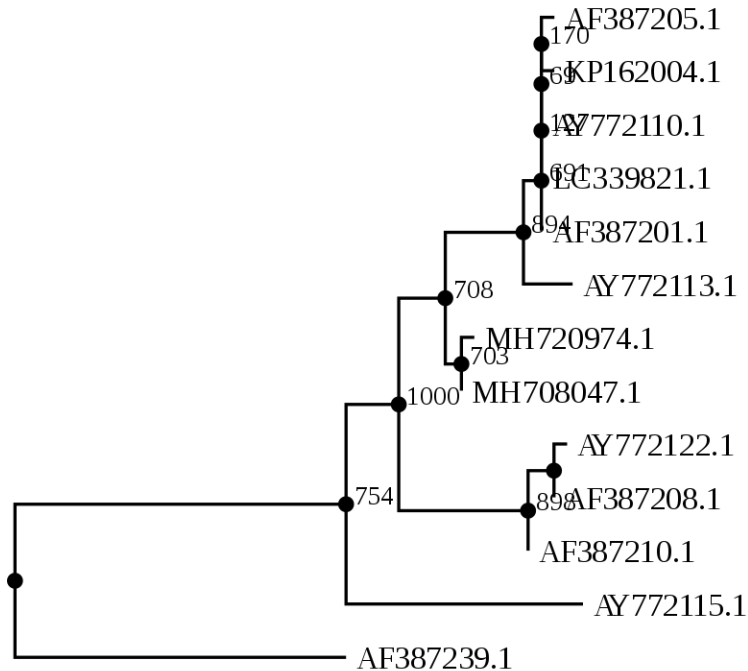

Supplement: Supplementary material 1 [file zookeys-880-001-s001.pdf]
